# Analysis of ocular biometrics in cataract patients aged 40 and above in Suzhou, China

**DOI:** 10.3389/fmed.2026.1915104

**Published:** 2026-09-15

**Authors:** Yanfeng Zeng, Wei Song, Quan Yuan, Xiaoqin Wang, Yana Ma, Huan Hu

**Affiliations:** 1 Li Xiang Eye Hospital, Soochow University, Suzhou, China; 2 Department of Child and Adolescent Health and Social Medicine, School of Public Health, Suzhou Medical College of Soochow University, Suzhou, China; 3 Suzhou Medical College of Soochow University, Suzhou, China; 4 Department of Ophthalmology, Xishui Hospital, Hubei University of Science and Technology, Xianning, China

**Keywords:** anterior chamber depth, axial length, cataracts, intraocular, ocular biometrical parameters

## Abstract

**Purpose:**

This cross-sectional study investigated the distribution of ocular biometric parameters in cataract patients aged ≥40 years from East China, with a specific focus on age- and sex-related differences and their correlations. Given the paucity of large-sample reference data for this population, we aimed to establish a localized framework to support precise surgical planning in refractive cataract surgery.

**Methods:**

This retrospective study consecutively enrolled patients aged ≥40 years who underwent cataract surgery at Lixiang Eye Hospital of Soochow University between January 2019 and September 2022. Axial length (AL), anterior chamber depth (ACD), corneal curvature (K1, K2), corneal astigmatism, intraocular lens (IOL) power, and intraocular pressure (IOP) were assessed. Non-parametric tests, Spearman correlation analyses, and multivariable linear regression were applied using SPSS 22.0 (IBM Corp.).

**Results:**

A total of 3,311 cataract patients (1,458 males, 1,853 females) were included. Female eyes had shorter AL and shallower ACD, but steeper corneal curvatures (K1, K2) compared to male eyes (all *P* < 0.001). With-the-rule astigmatism was significantly more prevalent in females, whereas against-the-rule astigmatism predominated in males. AL and ACD decreased with advancing age, and IOP was also negatively correlated with age. Corneal curvatures K1 and K2 increased with age, while corneal astigmatism showed a U-shaped pattern: initially decreasing before rising again after age 60–69. AL was positively correlated with ACD and negatively correlated with corneal curvature. Multivariable regression confirmed that age and sex were independently associated with all examined biometric parameters (all *P* < 0.05). Quantitatively, each additional year of age was associated with a 0.080 mm decrease in AL and a 0.182 D increase in IOL power.

**Conclusion:**

This study provides comprehensive, large-sample reference data on ocular biometric parameters for cataract patients in East China. We identify distinct age- and sex-specific patterns, particularly the non-linear age-related changes in corneal astigmatism and the sex-specific differences in astigmatism axis distribution. These findings offer actionable benchmarks for preoperative IOL selection, astigmatism management strategies, and patient counseling, thereby contributing to optimized refractive outcomes in cataract surgery.

## Introduction

Cataract is the leading cause of reversible blindness worldwide, severely impacting the quality of life and visual health of middle-aged and elderly populations (1). With the accelerating global aging process, its prevalence continues to incrise, making it a major public health concern (2). Currently, surgery is recognized as the only effective treatment, wherein the opaque crystalline lens is removed and an intraocular lens (IOL) is implanted, allowing patients to rapidly and safely regain visual function (3). In recent years, with advances in phacoemulsification techniques and the advent of premium IOLs, patient expectations for postoperative visual quality have fundamentally shifted (4). The surgical goal has evolved from traditional “vision restoration” to achieving clear, comfortable, and full-range refractive visual outcomes (5, 6). This paradigm shift places much higher demands on preoperative biometry, IOL power calculation, corneal astigmatism management, and surgical planning (7).

Ocular biometric parameters—including axial length (AL), anterior chamber depth (ACD), keratometry (K1/K2), and corneal astigmatism—are core input variables for IOL calculation formulas and are critical for predicting postoperative refractive outcomes, avoiding refractive surprises, and guiding astigmatism correction (8–10). Additionally, intraocular pressure (IOP) was also measured as a clinically relevant parameter for comprehensive patient evaluation, though it is not directly used in IOL power calculation. Previous studies have demonstrated that these biometric characteristics vary significantly by race, geography, sex, and age (11–13). Distinct differences in AL and keratometry distributions exist between Western and Asian populations, and even among different regions of China, middle-aged and elderly populations exhibit unique biological features (14–16).

However, despite these known variations, there remains a notable paucity of large-sample biometric studies specifically targeting cataract patients aged 40 and above in East China. Existing research has often focused on either general population-based surveys (11, 17) or specific myopic subgroups (16), leaving a gap in precise, localized reference data for routine clinical practice in this population. Furthermore, few studies have systematically analyzed the combined effects and interactions of age and sex across a comprehensive panel of biometric parameters within a single, large-scale surgical cohort (5, 6, 8–10). Most previous reports relied on bivariate correlation analyses without employing multivariable regression to determine whether observed associations were independent or confounded by other factors (10, 18).

This study provides several novel contributions beyond confirming known trends. To our knowledge, it represents the largest biometry study (3,311 eyes) specifically focused on this East Chinese population. Unlike previous reports that often analyzed isolated parameters or employed heterogeneous surgical protocols, we systematically evaluated the combined distribution of multiple inter-correlated parameters within a homogeneous cohort treated with monofocal non-toric IOLs without active astigmatism correction. This design allows for an unbiased description of natural biometric relationships. Notably, we identified a non-linear (U-shaped) age-related pattern in corneal astigmatism magnitude with an inflection point at 60–69 years—a finding not previously delineated in Chinese cataract cohorts—and quantified age- and sex-specific distributions of astigmatism axis, providing actionable benchmarks for preoperative counseling and surgical planning (19). Furthermore, we employed multivariable linear regression to determine whether age and sex are independently associated with each biometric parameter, extending beyond the bivariate correlation analyses reported in previous studies. This methodological enhancement allows us to quantify the independent contribution of age and sex, providing more robust evidence for clinical reference.

Based on 3,311 consecutive cataract surgery cases at Lixiang Eye Hospital of Soochow University, this study systematically analyses the distribution characteristics of AL, ACD, keratometry, corneal astigmatism, IOP, and IOL power. We specifically explore sex- and age-related differences, as well as the correlations between these parameters, aiming to provide localized, large-sample reference data for precise surgical planning, IOL selection, and astigmatism management in refractive cataract surgery.

## Materials and methods

### Subjects

This retrospective study consecutively enrolled patients aged 40 years or older who underwent cataract surgery at Lixiang Eye Hospital of Soochow University between January 2019 and September 2022.

Inclusion criteria were: (1) clinical diagnosis of cataract with subsequent cataract surgery at our institution; (2) complete preoperative biometric data available; and (3) patients or their legal guardians provided written informed consent for the use of clinical data for research purposes.

Exclusion criteria were: (1) presence of other concurrent ocular diseases (e.g., glaucoma, uveitis, corneal diseases, retinal pathologies, or optic nerve disorders); (2) history of prior ocular trauma or surgery; (3) severe systemic organ dysfunction; (4) active systemic infections; (5) severe mental health or consciousness disorders that precluded cooperation with examinations.

All patients underwent routine preoperative ophthalmological examinations. Because this study utilized only anonymized objective clinical data and did not involve personally identifiable information, the requirement for written informed consent from participants was waived. The study protocol was approved by the Medical Ethics Committee of Lixiang Eye Hospital of Soochow University (Approval No: SLER2024102) and adhered to the principles of the Declaration of Helsinki.

### IOL type selection and surgical protocol

All patients were implanted with conventional monofocal aspheric IOLs (including models such as SOFTEC, A1-UV, A2, ALD, IQ, PCL, and AQBH). Toric IOLs were not used. IOL power was calculated using formulas integrated into the IOLMaster 700 device. Formula selection was guided by axial length according to established clinical practice: the SRK/T formula was used for eyes with normal axial length (22.0 mm ≤ AL < 25.5 mm); the Barrett Universal II formula was preferred for short eyes (AL < 22.0 mm) and long eyes (AL ≥ 25.5 mm) based on its demonstrated superior predictive accuracy in these subgroups (10, 15, 20, 21); for eyes with extreme axial lengths, the Haigis formula was also employed. Target refraction was set to emmetropia or slight myopia (within −0.50 D to 0.00 D) based on each patient's individual visual needs and occupational requirements. The IOL power closest to this target was selected.

Regarding astigmatism management, no active astigmatism correction procedures were performed intraoperatively. Specifically, we did not implant Toric IOLs, perform limbal relaxing incisions (LRI), or apply femtosecond laser astigmatic keratotomy. The only intraoperative adjustment for preexisting corneal astigmatism was a minor optimization of the main corneal incision site, placed according to each surgeon's routine clinical practice. This standardized approach accurately reflects the most common clinical protocol at our center during the study period and ensures that the measured biometric relationships are not confounded by active astigmatism interventions.

### Ocular biometric measurements

All ocular biometric parameters were measured using the IOLMaster 700 swept-source optical coherence tomography biometer (Carl Zeiss Meditec AG, Jena, Germany) (22). The following parameters were recorded for each patient: axial length (AL), anterior chamber depth (ACD), flat corneal curvature (K1), steep corneal curvature (K2), mean corneal curvature, and corneal astigmatism magnitude (calculated as the difference between K2 and K1). Corneal astigmatism axis was classified into three categories based on the orientation of the negative cylinder axis: with-the-rule astigmatism (WTR, 0–30° or 150–180°), against-the-rule astigmatism (ATR, 60–120°), and oblique astigmatism (30–60° or 120–150°).

Intraocular pressure (IOP) was measured using a non-contact tonometer (*N*T-510, Nidek Co., Ltd., Gamagori, Japan). All measurements were performed by trained ophthalmic technicians under standardized conditions to ensure reproducibility. Notably, IOP was included as an additional clinical parameter for comprehensive patient assessment but is not a component of any IOL power calculation formula.

### Age and parameter grouping

Patients were stratified into five age groups at 10-year intervals: 40–49, 50–59, 60–69, 70–79, and ≥80 years. IOL power was categorized into six groups: <0 D, 0.0–5.9 D, 6.0–11.9 D, 12.0–17.9 D, 18.0–23.9 D, and ≥24.0 D. AL was classified into five groups: <22.00 mm, 22.00–23.99 mm, 24.00–25.99 mm, 26.00–27.99 mm, and ≥28.00 mm.

### Statistical analysis

Statistical analyses were performed using SPSS version 22.0 (IBM Corp., Armonk, NY, USA). The normality of distribution for all continuous variables was assessed using the Kolmogorov–Smirnov test. Since all variables showed non-normal distributions (*P* < 0.05), data are presented as medians with interquartile ranges (P25, P75). Accordingly, non-parametric methods were applied throughout: the Mann–Whitney *U*-test was used for comparisons between two independent groups (e.g., males vs. females), and the Kruskal–Wallis test was used for comparisons among multiple groups (e.g., age groups). The chi-square (*χ*^2^) test was employed to compare the distribution of categorical variables (astigmatism axis categories) between groups.

Spearman's rank correlation coefficient was calculated to evaluate the bivariate relationships among age, AL, ACD, K1, K2, mean corneal curvature, corneal astigmatism, IOP, and IOL power.

To determine whether age and sex were independently associated with each biometric parameter after adjusting for potential confounding, multivariable linear regression analyses were performed. Separate regression models were constructed for each dependent variable (AL, ACD, K1, K2, mean corneal curvature, corneal astigmatism, and IOL power), with age (continuous) and sex (categorical, female vs. male) as independent variables. The coefficient of determination (*R*^2^), adjusted *R*^2^, and overall model *F*-tests were reported. A two-sided *P*-value of less than 0.05 was considered statistically significant.

## Results

### Patient demographics and ophthalmic biometry

A total of 3,311 cataract patients were enrolled, including 1,458 males and 1,853 females. Among all cases, 1,617 were left eyes and 1,694 were right eyes. The mean age was 71.80 years (range: 40 to 98 years).

The median AL was 23.40 (22.76, 24.31) mm (range: 19.69 to 35.88 mm); the median ACD was 3.08 (2.75, 3.40) mm (range: 1.88 to 5.37 mm); the median mean corneal curvature was 44.38 (43.25, 45.56) D (range: 35.75 to 51.81 D); the median flat corneal curvature K1 was 44.00 (42.75, 45.12) D (range: 35.12 to 51.00 D); the median steep corneal curvature K2 was 44.87 (43.68, 46.00) D (range: 36.25 to 53.00 D); the median corneal astigmatism was −0.75 (−1.25, −0.38) D (range: 0.00 to −9.12 D); the median IOP was 13.70 (11.70, 16.00) mmHg (range: 5.70 to 22.70 mmHg); and the median IOL power was 20.50 (18.50, 22.00) D (range: −7.00 to 33.00 D). Regarding the axis distribution of corneal astigmatism, 44.7% of patients had with-the-rule astigmatism, 36.2% had against-the-rule astigmatism, and 19.1% had oblique astigmatism.

### Comparison of ocular biometric parameters across genders

Comparisons of AL, ACD, mean corneal curvature, corneal curvature (K1 and K2), corneal astigmatism, IOP, and IOL power between genders are presented in Table 1. No statistically significant difference was observed in IOP between males and females (*P* = 0.472). However, there were statistically significant differences between the two genders in AL, ACD, mean corneal curvature, corneal curvature (K1 and K2), corneal astigmatism, and IOL power (all *P* < 0.001). Compared with male patients, females had shorter AL, shallower ACD, higher mean corneal curvature and corneal astigmatism, larger corneal curvature (K1 and K2), and higher IOL power.

**Table 1:** Comparison of ocular biometric parameters in cataract patients of different genders.

Gender	Number of cases	Axial length (mm)	Anterior chamber depth (mm)	Diopter of intraocular lens (D)	Corneal curvature (D)		Mean corneal curvature (D)	Corneal astigmatism (D)	Intraocular pressure (mmHg)
					K1	K2			
Male	1,458	23.67 (23.06, 24.51)	3.18 (2.86, 3.47)	20.50 (18.50, 21.50)	43.50 (42.37, 44.50)	44.25 (43.25, 45.37)	43.87 (42.88, 45.00)	−0.75(−1.13, −0.37)	13.70 (11.70, 16.00)
Female	1,853	23.13 (22.53, 24.09)	3.00 (2.69, 3.32)	21.00 (18.50, 22.50)	44.37 (43.25, 45.50)	45.25 (44.12, 46.37)	44.81 (43.69, 45.94)	−0.75(−1.25, −0.50)	14.00 (11.85, 16.00)
*Z*	—	−12.405	−9.460	−6.512	−14.301	−15.548	−15.182	−3.287	−0.719
*P*	—	<0.001	<0.001	<0.001	<0.001	<0.001	<0.001	0.001	0.472

K1, flat corneal curvature; K2, steep corneal curvature; D, diopter.

The distribution of the axis of corneal astigmatism between the two genders is shown in Table 2. The proportions of with-the-rule astigmatism and oblique astigmatism were higher in females than in males, while the proportion of against-the-rule astigmatism was lower in females than in males; the difference was statistically significant (*χ*^2^ = 7.98, *P* = 0.018).

**Table 2:** Comparison of the distribution of corneal astigmatism axis in cataract patients of different genders.

Gender	Distribution of astigmatism axis [number of cases (%)]			
	With-the-rule astigmatism	Oblique-axis astigmatism	Against-the-rule astigmatism	Total number of cases
Male	637 (43.7)	257 (17.6)	564 (38.7)	1,458
Female	842 (45.4)	376 (20.3)	635 (34.3)	1,853
*χ* ^2^	7.980			
*P*	0.018			

### Comparison of ocular biometric parameters across different age groups

Comparisons of AL, ACD, mean corneal curvature, corneal curvature (K1 and K2), corneal astigmatism, IOP, and IOL power across different age groups are shown in Table 3. The age-related trends of each parameter are visualized in Figure 1.

**Table 3:** Comparison of ocular biometric parameters of different age groups.

Ocular biometry		40–49	50–59	60–69	70–79	≥80	H	P
*N*		80	274	806	1,474	677		
Axial length (mm)		27.57 (24.59, 30.14)	24.40 (23.16, 27.91)	23.42 (22.76, 24.36)	23.33 (22.75, 24.12)	23.26 (22.68, 23.96)	195.608	<0.001
Anterior chamber depth (mm)		3.44 (3.16, 3.76)	3.30 (3.02, 3.61)	3.13 (2.79, 3.43)	3.05 (2.75, 3.37)	2.97 (2.66, 3.26)	151.912	<0.001
Diopter of intraocular lens (D)		11.00 (5.00, 19.38)	16.75 (8.00, 21.50)	20.50 (18.00, 22.00)	20.50 (19.00, 22.00)	21.00 (19.50, 22.00)	133.270	<0.001
Corneal curvature (D)	K1	42.75 (41.50, 43.84)	43.50 (42.62, 44.87)	44.00 (42.87, 45.12)	44.00 (42.97, 45.12)	44.00 (42.75, 45.12)	47.717	<0.001
	K2	43.87 (42.53, 45.12)	44.50 (43.37, 45.75)	44.75 (43.75, 45.87)	45.00 (43.75, 46.12)	45.00 (43.75, 46.25)	46.513	<0.001
Mean corneal curvature (D)		43.09 (41.92, 44.48)	44.00 (43.00, 45.20)	44.38 (43.30, 45.50)	44.50 (43.37, 45.56)	44.50 (43.37, 45.75)	46.633	<0.001
Corneal astigmatism (D)		−1.12(−1.50, −0.50)	−0.75(−1.25, −0.38)	−0.63(−1.12, −0.37)	−0.75(−1.13, −0.38)	−1.00(−1.50, −0.50)	65.931	<0.001
Intraocular pressure (mmHg)		15.15 (12.85, 16.70)	14.00 (12.60, 16.00)	14.00 (12.00, 16.30)	13.70 (11.70, 15.78)	13.30 (11.30, 15.30)	37.983	<0.001

K1, flat corneal curvature; K2, steep corneal curvature; D, diopter.

**Figure 1:**
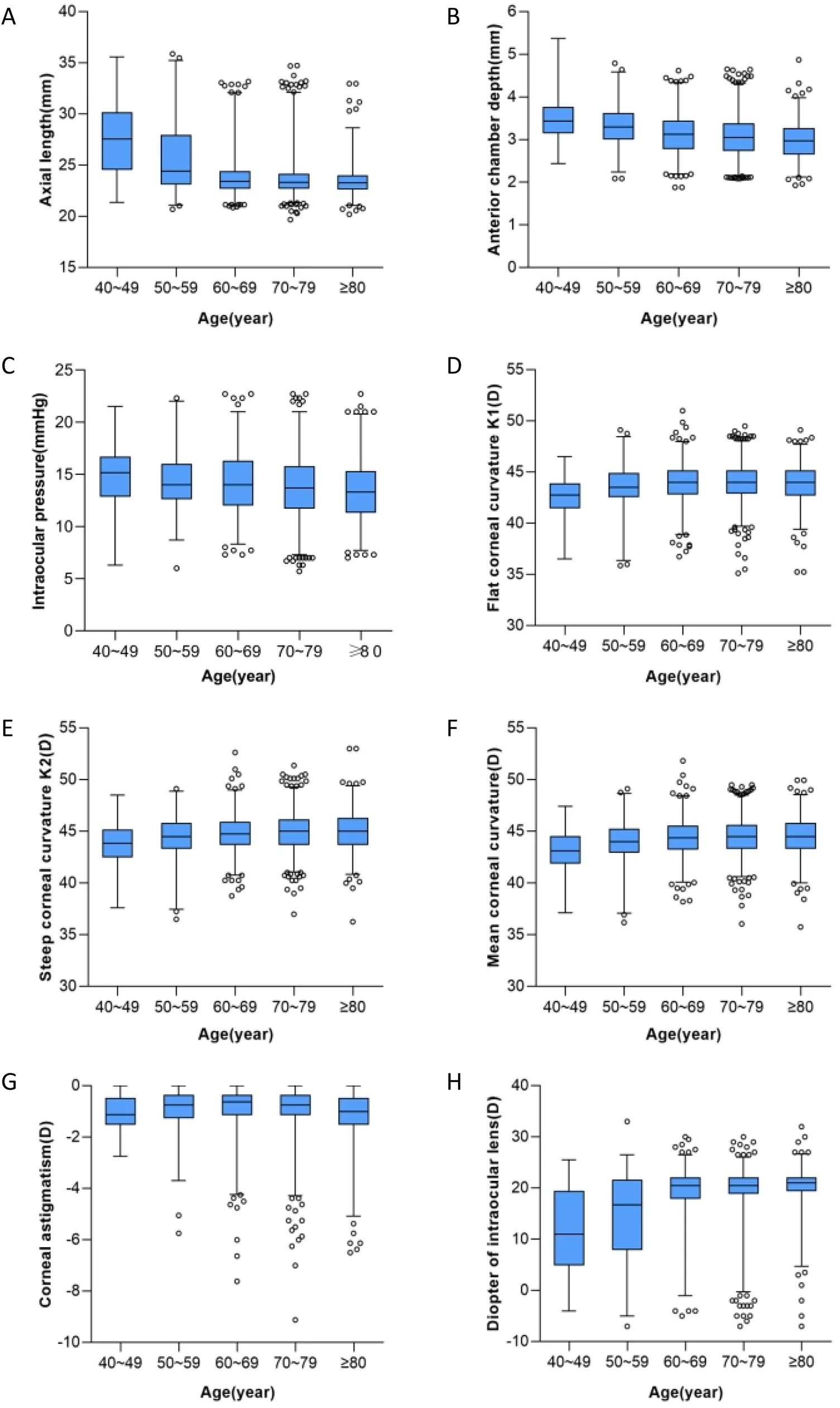
Age-related trends in ocular biometric parameters. Box plots showing the distribution of **(A)** axial length, **(B)** anterior chamber depth, **(C)** IOL power, **(D)** flat corneal curvature K1, **(E)** steep corneal curvature K2, **(F)** mean corneal curvature, **(G)** corneal astigmatism, and **(H)** intraocular pressure across five age groups (40–49, 50–59, 60–69, 70–79, and ≥80 years). Boxes represent the interquartile range (IQR), horizontal lines indicate medians, and whiskers extend to the 5th and 95th percentiles. D, diopter; mmHg, millimeters of mercury. Statistical comparisons are provided in Table 3.

Age was stratified at 10-year intervals. The results revealed that AL, ACD, and IOP exhibited a decreasing trend with increasing age. In contrast, IOL power, mean corneal curvature, horizontal corneal curvature K1, and vertical corneal curvature K2 showed an increasing trend, while corneal astigmatism initially decreased and then increased, with the inflection point occurring between 60 and 69 years of age. The differences were statistically significant (all *P* < 0.001).

The comparison of the distribution of corneal astigmatism axis across different age groups is shown in Table 4. As age increased, the proportion of with-the-rule astigmatism decreased, while the proportion of against-the-rule astigmatism increased. The proportion of oblique astigmatism remained relatively stable; the difference was statistically significant (*χ*^2^ = 190.993, *P* < 0.001). The shift in astigmatism axis distribution with increasing age is shown in Figure 2.

**Table 4:** Comparison of the distribution of corneal astigmatism axis among different age groups.

Age group (years)	Axis distribution [number of cases (%)]			
	With-the-rule astigmatism	Oblique-axis astigmatism	Against-the-rule astigmatism	Total number of cases
40–49	67 (83.8)	7 (8.8)	6 (7.5)	80
50–59	170 (62.0)	55 (20.1)	49 (17.9)	274
60–69	428 (53.1)	156 (19.4)	222 (27.5)	806
70–∼79	600 (40.7)	283 (19.2)	581 (40.1)	1,474
≥80	214 (31.6)	132 (19.5)	331 (48.9)	677
*χ* ^2^	190.993			
*P*	<0.001			

**Figure 2:**
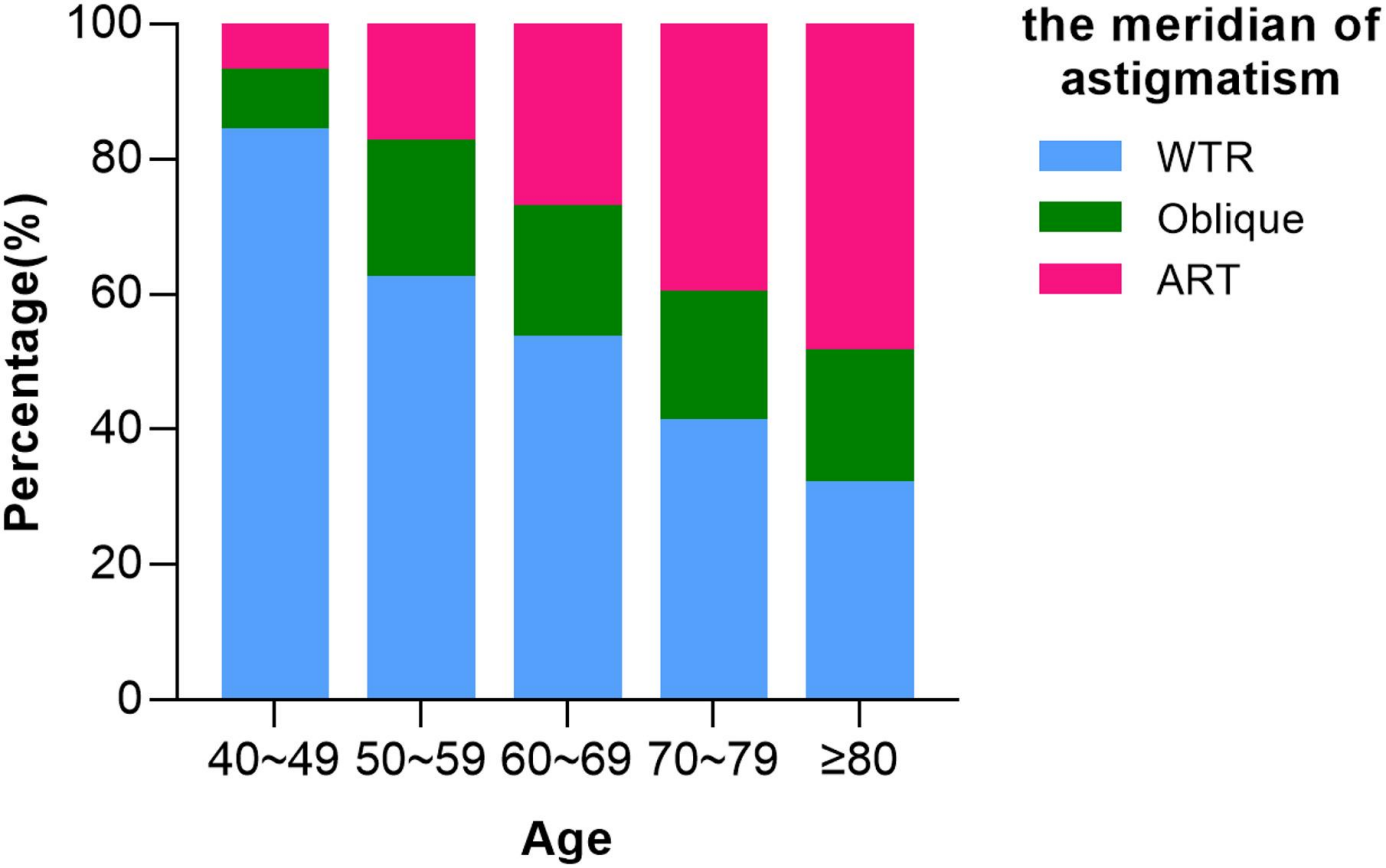
Age-related shift in corneal astigmatism axis distribution. Stacked bar chart showing the proportion of with-the-rule (blue), oblique (gray), and against-the-rule (orange) astigmatism across five age groups (40–49, 50–59, 60–69, 70–79, and ≥80 years). The proportion of with-the-rule astigmatism decreased progressively with age, whereas against-the-rule astigmatism increased, reflecting a significant shift in astigmatism axis with advancing age (*χ*^2^ = 190.993, *P* < 0.001). Numerical data are provided in Table 4.

### Correlation analysis

Significant correlations were observed among age, AL, ACD, IOL power, K1, K2, corneal astigmatism, and IOP, as detailed in Table 5. Specifically, age was positively correlated with IOL power, K1, and K2 (*r* = 0.294, 0.095, and 0.124, respectively; all *P* < 0.001). However, age was negatively correlated with AL, ACD, corneal astigmatism, and IOP (*r* = −0.328, −0.219, −0.057, and −0.109, respectively; all *P* ≤ 0.001).

**Table 5:** Correlation analysis between various ocular biometric parameters.

Variable	Statistic	Age	Axial length	Anterior chamber depth	Diopter of intraocular lens	Flat corneal curvature K1)	Steep corneal curvature K2	Mean corneal curvature	Corneal astigmatism	Intraocular pressure
Age	*r*	1.000								
	*P*									
Axial length	*r*	−0.328**	1.000							
	*P*	<0.001								
Anterior chamber depth	*r*	−0.219**	0.367**	1.000						
	*P*	<0.001	<0.001							
Diopter of intraocular lens	*r*	0.294**	−0.930**	−0.359**	1.000					
	*P*	<0.001	<0.001	<0.001						
Flat corneal curvature K1	*r*	0.095**	−0.330**	−0.121**	0.096**	1.000				
	*P*	<0.001	<0.001	<0.001	<0.001					
Steep corneal curvature K2	*r*	0.124**	−0.294**	−0.135**	0.043*	0.888**	1.000			
	*P*	<0.001	<0.001	<0.001	0.014	<0.001				
Mean corneal curvature	*r*	0.113**	−0.321**	−0.132**	0.072**	0.972**	0.971**	1.000		
	*P*	<0.001	<0.001	<0.001	<0.001	<0.001	<0.001			
Corneal astigmatism	*r*	−0.057**	−0.086**	0.026	0.115**	0.268**	−0.205**	0.034*	1.000	
	*P*	0.001	<0.001	0.128	<0.001	<0.001	<0.001	0.049		
Intraocular pressure	*r*	−0.109**	0.101**	0.021	−0.108**	0.035*	0.007	0.022	0.060**	1.000
	*P*	<0.001	<0.001	0.232	<0.001	0.042	0.676	0.206	0.001	

* *P* < 0.05; **P < 0.01.

AL showed a positive correlation with ACD and IOP (*r* = 0.367 and 0.101, respectively; both *P* < 0.001), and a negative correlation with IOL power, K1, K2, and corneal astigmatism (*r* = −0.930, −0.330, −0.294, and −0.086, respectively; all *P* < 0.001).

ACD was negatively correlated with IOL power, K1, and K2 (*r* = −0.359, −0.121, and −0.135, respectively; all *P* < 0.001), but showed no correlation with IOP (*P* = 0.232).

K1 was positively correlated with IOL power, K2, and IOP (*r* = 0.096, 0.888, and 0.035, respectively; all *P* ≤ 0.042). K2 was positively correlated with IOL power (*r* = 0.043, *P* = 0.014) and negatively correlated with AL (*r* = −0.294, *P* < 0.001). Corneal astigmatism was positively correlated with IOP (*r* = 0.060, *P* = 0.001). Correlation scatter plots among ocular biometric parameters are presented in Figure 3.

**Figure 3:**
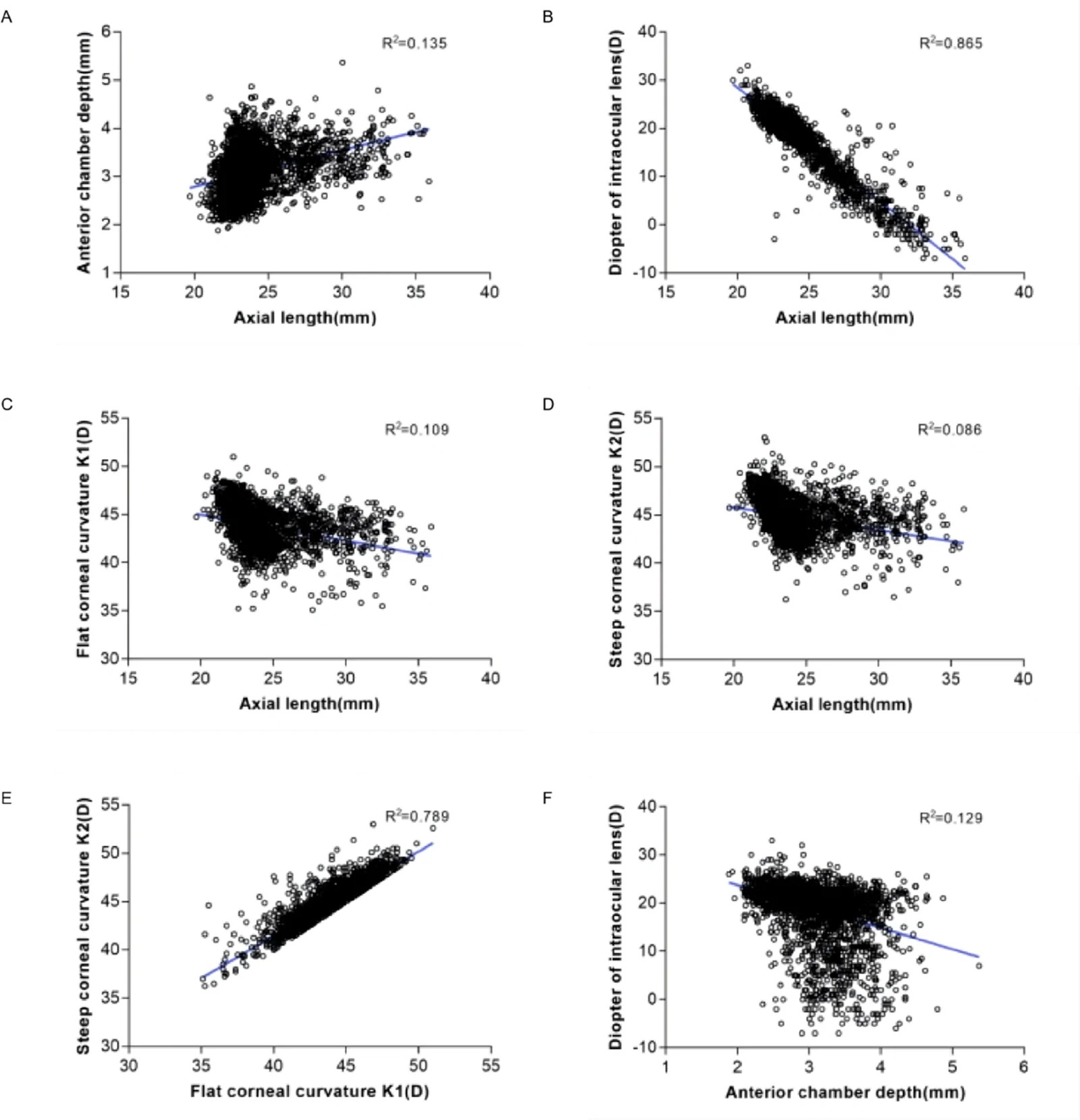
Correlation scatter plots among ocular biometric parameters. Scatter plots showing pairwise correlations between selected parameters: **(A)** axial length (AL) vs. anterior chamber depth (ACD); **(B)** AL vs. intraocular lens (IOL) power; **(C)** AL vs. flat corneal curvature (K1); **(D)** AL vs. steep corneal curvature (K2); **(E)** steep corneal curvature (K2) vs. flat corneal curvature (K1); **(F)** IOL power vs. ACD. Each point represents an individual eye. The regression line and 95% confidence intervals are shown in blue. Correlation coefficients (Spearman's *r*) and *P* values are provided in Table 5.

The age group-specific correlation patterns are visualized in Figure 4. For patients aged 40–49 years, only ACD and IOL power showed weak negative correlations with age (*r* = −0.223, *P* = 0.047 for ACD; *r* = −0.241, *P* = 0.031 for IOL power), whereas no significant correlations were found for the other biometric parameters (all *P* > 0.05). In contrast, patients aged 50–59 showed positive correlations with IOL power, K1, and K2 (*r* = 0.184, 0.197, and 0.185, respectively; all *P* < 0.01), and negative correlations with AL and ACD (*r* = −0.209 and −0.128, respectively; both *P* < 0.05). The 60–69 age group showed a positive correlation with IOL power (*r* = 0.114, *P* < 0.01). The 70–79 age group showed a negative correlation with ACD (*r* = −0.089, *P* < 0.01).

**Figure 4:**
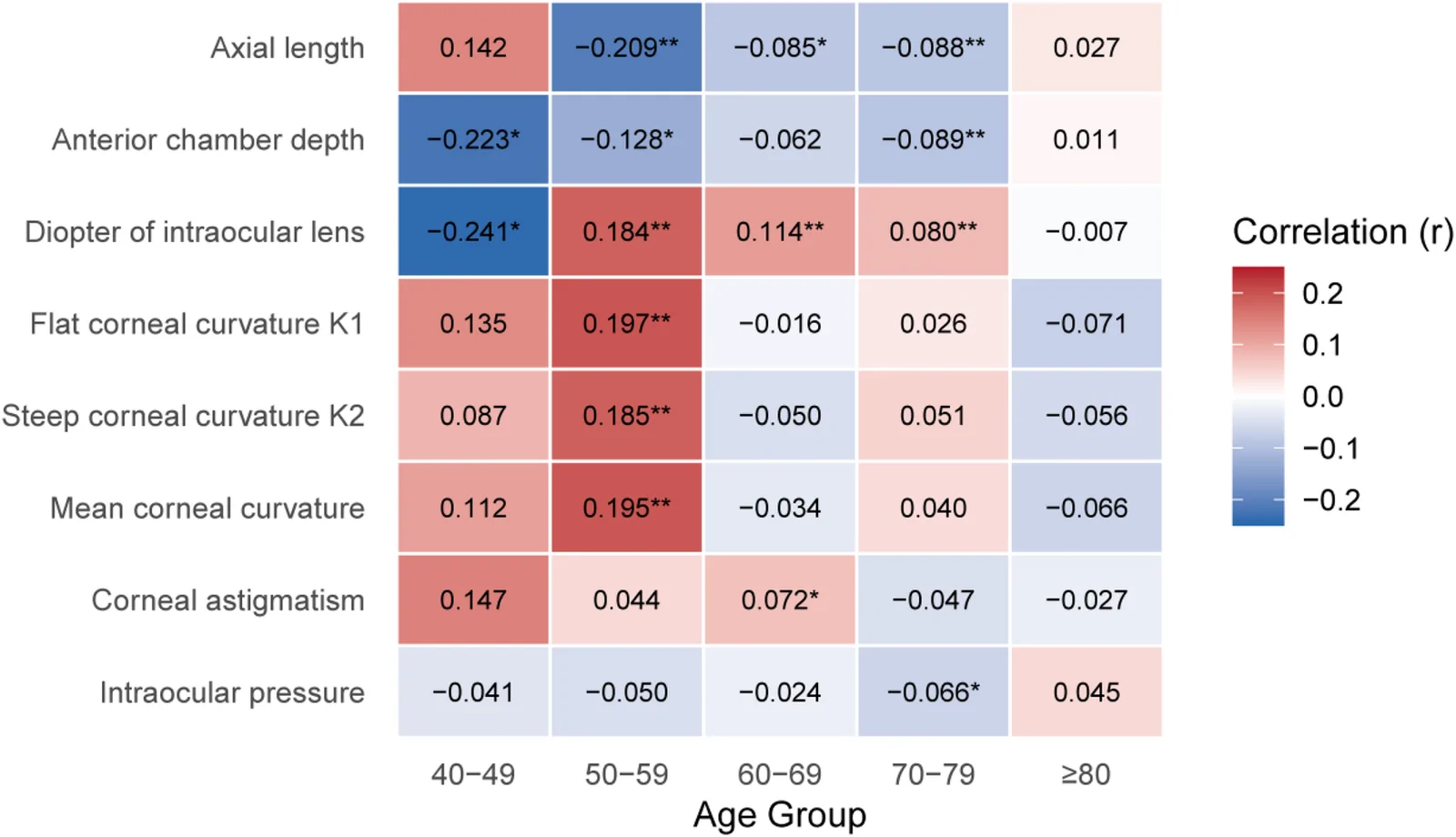
Correlation heatmap between age groups and ocular biometric parameters. The color intensity represents the Spearman correlation coefficient (*r*), with red indicating positive correlations and blue indicating negative correlations. Cell values show the correlation coefficients, with * denoting *P* < 0.05 and ** denoting *P* < 0.01. AL, axial length; ACD, anterior chamber depth; IOL, intraocular lens; K1, flat corneal curvature; K2, steep corneal curvature; IOP, intraocular pressure.

### Multivariable regression analysis

To further evaluate the independent effects of age and sex on each biometric parameter, multivariable linear regression analyses were performed. As shown in Table 6, after adjusting for the other variable, both age and sex remained independently associated with all examined parameters (all *P* < 0.05), except for the sex effect on IOP (*P* = 0.472, consistent with the univariate comparison).

**Table 6:** Multivariable linear regression analysis of the effects of age and sex on ocular biometric parameters.

Outcome	Predictor	*B*	SE	*t*	*P*	CI lower	CI upper	*R* ^2^	Adj-*R*^2^	*F*	*P*	*N*
Axial length (mm)	Age	−0.0797	0.0039	−20.199	<0.001	−0.0875	−0.072					
	Sex (female vs. male)	−0.5046	0.0739	−6.829	<0.001	−0.6495	−0.3597					
								0.120	0.119	225.486	<0.001	3,311
Anterior chamber depth (mm)	Age	−0.0111	0.0008	−13.259	<0.001	−0.0127	−0.0095					
	Sex (female vs. male)	−0.1546	0.0157	−9.869	<0.001	−0.1853	−0.1239					
								0.075	0.075	134.855	<0.001	3,311
Flat K1 (D)	Age	0.0194	0.0033	5.849	<0.001	0.0129	0.026					
	Sex (female vs. male)	0.8521	0.0622	13.699	<0.001	0.7302	0.9741					
								0.062	0.062	109.866	<0.001	3,311
Steep K2 (D)	Age	0.0248	0.0032	7.659	<0.001	0.0184	0.0311					
	Sex (female vs. male)	0.9375	0.0606	15.473	<0.001	0.8187	1.0563					
								0.082	0.081	147.460	<0.001	3,311
Mean corneal curvature (D)	Age	0.0221	0.0032	6.954	<0.001	0.0159	0.0284					
	Sex (female vs. male)	0.8948	0.0595	15.031	<0.001	0.7781	1.0116					
								0.076	0.075	135.749	<0.001	3,311
Corneal astigmatism (D)	Age	−0.005	0.0016	3.331	0.001	−0.009	−0.002					
	Sex (female vs. male)	−0.085	0.0301	2.838	0.005	−0.144	−0.026					
								0.006	0.005	9.449	<0.001	3,311
IOL power (D)	Age	0.182	0.0102	17.767	<0.001	0.1619	0.2021					
	Sex (female vs. male)	0.4733	0.1918	2.468	0.014	0.0973	0.8493					
								0.088	0.088	160.317	<0.001	3,311

Multivariate linear regression models were built for each ocular biometric outcome to evaluate the independent effects of age and sex, with mutual adjustment between the two predictors. Male participants were set as the reference group for sex. Corneal astigmatism was analyzed using raw values (with axis direction, where with-the-rule astigmatism is represented as a negative value). All statistical tests were two-sided. B, unstandardized regression coefficient; SE, standard error; CI, confidence interval; IOL, intraocular lens; K1, flat keratometry; K2, steep keratometry.

Specifically, for every 1-year increase in age, AL decreased by 0.080 mm (*β* = −0.0797, 95% CI: −0.0875 to −0.0720, *P* < 0.001), ACD decreased by 0.011 mm (*β* = −0.0111, 95% CI: −0.0127 to −0.0095, *P* < 0.001), and IOL power increased by 0.182 D (*β* = 0.1820, 95% CI: 0.1619 to 0.2021, *P* < 0.001). Female sex was independently associated with shorter AL (*β* = −0.5046, 95% CI: −0.6495 to −0.3597, *P* < 0.001), shallower ACD (*β* = −0.1546, 95% CI: −0.1853 to −0.1239, *P* < 0.001), steeper K1 (*β* = 0.8521, 95% CI: 0.7302 to 0.9741, *P* < 0.001), and steeper K2 (*β* = 0.9375, 95% CI: 0.8187 to 1.0563, *P* < 0.001), after adjusting for age. The regression models explained 0.5% to 11.9% of the variance in the respective outcomes (adjusted *R*^2^ range: 0.0051 for corneal astigmatism to 0.1194 for AL).

## Discussion

Cataract is the leading cause of blindness worldwide (23), and surgery remains the only effective treatment. With advancements in technology, patients now have higher demands and expectations. The purpose of cataract surgery has shifted from blindness prevention to refractive correction. Therefore, understanding the distribution of various ocular biometric parameters in patients before cataract surgery can provide valuable insights for improving postoperative visual quality.

In this large-sample cohort of 3,311 cataract patients from East China, we systematically characterized the distribution of key ocular biometric parameters and their correlations with age and sex. Our main findings are threefold: (1) significant sex differences exist in AL, ACD, corneal curvature, and astigmatism axis; (2) AL, ACD, and IOP decrease with age, while corneal curvatures increase; and (3) corneal astigmatism exhibits a non-linear U-shaped age-related pattern with an inflection point at 60–69 years. These findings, which are derived from a homogeneous surgical cohort without active astigmatism correction, provide region-specific reference data that directly inform surgical planning and patient counseling. Importantly, our multivariable regression analysis extends beyond prior bivariate correlation studies by confirming that both age and sex are independently associated with each biometric parameter, providing quantified estimates for clinical reference.

### Sex differences in ocular biometrics

The results of this study indicate that the AL and ACD are shorter in females than in males (median AL: 23.13 mm vs. 23.67 mm; median ACD: 3.00 mm vs. 3.18 mm; both *P* < 0.001). Studies by Shinojima et al. (10) and Huang et al. (5) have similarly found that women have shorter ALs and shallower ACDs compared to men. The observed differences in AL and ACD between genders are likely attributable to variations in height and body stature, as axial length is known to correlate positively with height (11, 12). This study also revealed that K1 and K2 in females are greater than those in males (median K2: 45.25 D vs. 44.25 D; median K1: 44.37 D vs. 43.50 D; both *P* < 0.001). Many previous studies have also yielded similar results (13, 24); this steeper corneal curvature in females partially compensates for their shorter axial length in terms of overall ocular refractive power. Our multivariable regression analysis confirmed that female sex remained an independent predictor of shorter AL, shallower ACD, and steeper corneal curvatures after adjusting for age (all *P* < 0.001), consistent with findings from the Beijing Eye Study (11).

A notable sex difference was observed in the distribution of corneal astigmatism axis. The proportion of female patients with with-the-rule astigmatism was higher than that of men (45.4% vs. 43.7%, respectively), and the proportion with against-the-rule astigmatism was lower than that of men (34.3% vs. 38.7%, respectively) (Table 2). This sex-specific axis distribution has practical implications: for female patients presenting with with-the-rule astigmatism, surgeons may consider under-correction of the cylinder, as the natural age-related shift toward against-the-rule astigmatism may partially reduce the refractive error over time. Conversely, for male patients with against-the-rule astigmatism, more precise correction may be warranted preoperatively.

### Age-related changes and non-linear patterns

In this study, we found that AL was shorter and ACD was shallower with increasing age. This is similar to the results of several studies in Israel (9), Australia (12), the United States (24), and southern China (6). The age-related decrease in AL may be explained by a cohort effect: younger patients with high myopia tend to undergo cataract surgery earlier, introducing a selection bias in this cross-sectional study. Moreover, the declining ACD with age is likely attributable to progressive lens thickening and forward displacement of the iris-lens diaphragm, a well-documented age-related physiological change (6). Our multivariable regression further quantified that each additional year of age was associated with a 0.080 mm decrease in AL and a 0.011 mm decrease in ACD, independent of sex.

Our finding that IOP was negatively correlated with age adds to the ongoing debate regarding the age-IOP relationship. While some Western studies have reported a positive association between age and IOP (18, 25–28), our results align with several Asian population-based surveys from South Korea (29, 30), Japan (31–33), China (34), and Taiwan (17), which similarly demonstrated a negative or neutral age-IOP relationship. This discrepancy is likely attributable to regional and racial differences, as well as variations in study design and measurement protocols. Importantly, although IOP is not a component of IOL calculation formulas, its age-related decline in this cataract population has clinical relevance: it reminds clinicians to monitor IOP changes in elderly cataract patients, particularly those with pre-existing glaucoma risk factors, both before and after surgery.

Analysis with age stratification indicated that as age increased, both horizontal corneal curvature K1 and vertical corneal curvature K2 exhibited an upward trend while corneal astigmatism initially decreased before increasing, with the turning point occurring at the age of 60 to 69 years (35, 36). Unlike previous studies that often assumed a linear age-related increase in corneal astigmatism, our data reveal a more complex U-shaped trajectory. The underlying mechanism may involve age-related changes in eyelid tension, which gradually decreases with aging, leading to a reduction in with-the-rule astigmatism (37); later, corneal structural changes—including altered biomechanical properties and progressive flattening in the horizontal meridian—may contribute to the subsequent increase in against-the-rule astigmatism (38, 39). This U-shaped pattern is consistent with recent population-based findings from Turkey (39), which demonstrated similar age-related variations in corneal geometry. It is noteworthy that significant differences appeared in the corneal astigmatism axis among different age groups (*χ*^2^ = 190.993, *P* < 0.001). As age increased, the proportion of with-the-rule astigmatism declined, while the proportion of against-the-rule astigmatism rose, reflecting long-term dynamic changes in corneal astigmatism. These findings have direct clinical implications: for younger cataract patients (40–59 years) with with-the-rule astigmatism, surgeons may adopt a more conservative approach to astigmatism correction, given the anticipated natural shift toward against-the-rule astigmatism with advancing age. In contrast, for older patients (≥70 years), the astigmatism pattern is more stable, and active correction—such as Toric IOL implantation or limbal relaxing incisions—may be more beneficial.

In addition, among the cataract patients aged over 40 included in this study, IOL power increased with age, particularly for those aged 50 to 69 years. This age-related increase in IOL power is primarily driven by the corresponding decrease in AL and the increase in corneal curvature, as reflected in the strong negative correlation between IOL power and AL (r = −0.930, *P* < 0.001). Our regression analysis confirmed that age independently contributed to IOL power increase (*β* = 0.182 D per year, *P* < 0.001), even after adjusting for sex.

### Correlations among parameters and interpretation of regression findings

The results of Spearman correlation analysis showed that AL was positively correlated with ACD and negatively correlated with corneal curvature, consistent with the findings of Shinojima et al. (10). A strong positive correlation between K1 and K2 was observed (r = 0.888, *P* < 0.001), consistent with Liu et al. (16), reflecting the natural coupling of the two corneal meridians. However, our finding that ACD was negatively correlated with age and positively correlated with AL differs from Liu et al. (16), who reported no significant relationship between ACD and age or AL. This discrepancy may be attributed to differences in study populations: Liu et al. focused on cataract patients with ultra-long AL, whereas our study included the general cataract patient population. The distinct biometric characteristics of highly myopic eyes—including deeper ACD and different lens positional relationships—may explain the divergent findings.

AL was positively correlated with IOP in our cohort (*r* = 0.101, *P* < 0.001). Lee et al. (40) suggested that high IOP may lead to eyeball enlargement, which can consequently result in elongated AL. Alternatively, longer eyes may have thinner scleras and altered biomechanical properties, potentially rendering them more susceptible to IOP elevation. Therefore, when performing surgery and treatment for cataract patients, surgeons should closely monitor IOP levels to adjust treatment approaches in order to achieve optimal outcomes and postoperative recovery. The negative correlation between AL and age in our cross-sectional data is likely influenced by a cohort effect: younger patients with high myopia typically undergo surgical intervention earlier, introducing an unavoidable selection bias. This limitation is inherent to the study design and should be considered when interpreting the age-related trends.

The relatively low *R*^2^ values observed in our regression models (ranging from 0.005 for corneal astigmatism to 0.119 for AL) indicate that age and sex together explain only a modest proportion of the variance in these biometric parameters. This is not unexpected, as ocular biometry is influenced by a multitude of factors including height, weight, genetic predisposition, educational level, and occupational exposure (11, 14, 15). Nevertheless, the independent associations we identified—with quantified *β* coefficients and 95% confidence intervals—provide clinically useful benchmarks that complement, rather than replace, comprehensive preoperative assessment. These quantified estimates allow surgeons to anticipate, on average, how much AL, ACD, and IOL power might vary with each decade of age and between sexes, thereby aiding in surgical planning and patient counseling.

### Clinical implications

Taken together, our findings offer several practical takeaways for cataract surgeons. First, the normative biometric data stratified by age and sex can serve as a reference for preoperative counseling, helping to set realistic patient expectations regarding expected biometric variations. Second, the U-shaped age-related pattern of corneal astigmatism and the shift in axis distribution with age provide evidence-based guidance for deciding whether and how aggressively to correct astigmatism at different ages. Third, the significant inter-sex differences in AL, ACD, and corneal curvature underscore the importance of sex-specific considerations in IOL power calculation and surgical planning. Fourth, the multivariable regression results provide quantified estimates of age and sex effects, as detailed in Table 6, enabling surgeons to anticipate expected biometric variations for individual patients based on their age and sex. Finally, the observed IOP-age relationship, while not directly influencing IOL selection, highlights the need for careful perioperative IOP monitoring, particularly in elderly patients with pre-existing glaucoma risk factors.

## Limitations

This study has several limitations that should be acknowledged. First, the cross-sectional design can only capture associations at a single time point; it cannot establish causality or distinguish between age-related changes and cohort effects. Longitudinal follow-up data are needed to confirm the dynamic trends we observed, particularly the U-shaped pattern of corneal astigmatism. Second, as a single-center study conducted in East China, our findings may not be generalizable to other regions within China or to different ethnic populations. Multi-center studies with broader geographic coverage are warranted. Third, our cohort consisted exclusively of patients who underwent cataract surgery, which may introduce selection bias, as patients with milder cataracts or those who declined surgery were not included. Fourth, we did not collect postoperative refractive outcomes or patient-reported visual quality measures, which would have allowed us to validate the clinical utility of our reference data. This is a direction for future research. Finally, although we performed multivariable regression, the relatively low *R*^2^ values (ranging from 0.005 to 0.119) indicate that age and sex explain only a modest proportion of the variance in these biometric parameters, and other unmeasured factors (e.g., height, weight, educational level, occupational exposure) may contribute significantly (11, 14, 15).

## Future directions

This study used the IOLMaster 700 to measure the main ocular biometric parameters in cataract patients aged 40 years and older. It describes the distribution of these parameters, focusing on age and sex differences, as well as their correlations, aiming to provide surgical guidance for cataract treatment. In future studies, we plan to collect postoperative refractive outcomes and compare them with preoperative predictions based on the biometric reference data established in this study. This will allow us to evaluate whether the age- and sex-specific benchmarks we identified translate into clinically meaningful differences in postoperative refractive accuracy, particularly in patients with extreme axial lengths or high astigmatism. Additionally, incorporating anthropometric measures such as height and weight into multivariable models would help further elucidate the mechanisms underlying sex differences in ocular biometry (11).

## Conclusion

This cross-sectional study of 3,311 cataract patients aged 40 years and older from East China provides comprehensive, large-sample reference data on ocular biometric parameters. Our findings demonstrate that AL, ACD, and corneal curvature exhibit significant sex- and age-related differences, with females having shorter AL, shallower ACD, and steeper corneal curvatures compared to males. Notably, we identified a non-linear (U-shaped) age-related pattern in corneal astigmatism magnitude, with an inflection point at 60–69 years, and a progressive shift from with-the-rule to against-the-rule astigmatism with advancing age. Multivariable regression confirmed that both age and sex are independently associated with all examined biometric parameters, providing quantified estimates for clinical reference (e.g., each additional year of age corresponds to a 0.080 mm decrease in AL and a 0.182 D increase in IOL power). These age- and sex-specific patterns offer actionable benchmarks for preoperative IOL selection, astigmatism management strategies, and patient counseling. While our cross-sectional design and single-center setting limit causal inference and generalizability, the data serve as a valuable reference for refractive cataract surgery planning in this population, and future longitudinal studies are warranted to validate the observed dynamic trends.

## Data Availability

The raw data supporting the conclusions of this article will be made available by the authors, without undue reservation.
